# Systematic Pan-Cancer Characterization of ST3GAL4 Reveals Its Prognostic and Immunologic Associations

**DOI:** 10.3390/biomedicines14040766

**Published:** 2026-03-27

**Authors:** Fushu Luo, Xiaoshun Sun, Changwu Wu, Jun Tan, Yimin Pan

**Affiliations:** 1 Department of Neurosurgery, Xiangya Hospital, Central South University, Changsha 410008, China; 228102120@csu.edu.cn (F.L.);; 2 National Clinical Research Center for Geriatric Disorders, Xiangya Hospital, Central South University, Changsha 410008, China; 3 Department of Critical Care Medicine, Xiangya Hospital, Central South University, Xiangya Road 87, Changsha 410008, China

**Keywords:** ST3GAL4, pan-cancer, glycosylation, prognostic marker, TCGA, immune infiltration

## Abstract

**Background:** Sialylation, a key terminal glycosylation modification, plays a pivotal role in tumor progression and immune evasion. The sialyltransferase ST3GAL4 is implicated in individual cancers, but its pan-cancer landscape and systemic associations remain undefined. **Methods:** We performed an integrated multi-omics analysis using transcriptomic, proteomic, genomic, DNA methylation, and tumor microenvironment datasets from TCGA, CPTAC, GTEx, and other public resources. Immune associations were evaluated via TIMER2.0 and TISIDB. Experimental validation included immunofluorescence staining for ST3GAL4 protein in human tumor specimens. **Results:** ST3GAL4 exhibited pervasive, lineage-specific dysregulation across cancers. Elevated expression correlated with adverse prognosis, genomic instability, and specific RNA modification patterns. Tumor microenvironment analyses revealed significant associations: ST3GAL4 expression positively correlated with cancer-associated fibroblast and endothelial cell infiltration but was inversely associated with cytotoxic T-cell abundance. Functional enrichment implicated ST3GAL4 within glycosphingolipid metabolism and glycan biosynthetic pathways. In experimental models, its expression demonstrated context-dependent modulation following cytokine stimulation and immunotherapy. Immunofluorescence confirmed tumor-specific protein expression and its spatial co-occurrence with stromal and immune cell markers. **Conclusions:** This multi-omics study delineates a comprehensive pan-cancer atlas of ST3GAL4, establishing its association with aggressive tumor behavior, an immunosuppressive microenvironment, and core glycosylation pathways. These findings position ST3GAL4 as a potential cross-tumor node linking sialylation to immune evasion, providing a rationale for future mechanistic and therapeutic exploration.

## 1. Introduction

Cancer remains one of the most heterogeneous and challenging diseases, driven by the interplay of genetic mutations, epigenetic reprogramming, dysregulated signaling pathways and microenvironmental interactions [1]. Beyond these canonical mechanisms, alterations at the protein level introduce an additional dimension of regulatory complexity to cancer biology [2]. As one of the most abundant post-translational modifications, glycosylation dynamically regulates protein stability, receptor signaling, and cell–cell communication in both physiological and pathological settings [3]. Among them, sialylation at the terminal ends of glycan chains therefore plays a crucial role in tumor progression [4]. Specifically, altered sialylation patterns have been linked to tumor aggressiveness, immune evasion, and therapeutic resistance across cancer types [4,5].

Among the four main sialyltransferase families (ST3GAL, ST6GAL, ST6GALNAC and ST8SIA), the beta-galactoside α-2,3-sialyltransferase (ST3GAL) family catalyzes the transfer of α-2,3-linked sialic acids onto glycoproteins and glycolipids, shaping the sialylation landscape of the cell surface [6,7]. This family comprises six members (ST3GAL1-6) that differ in substrate specificity and tissue distribution [7]. Aberrant expression of ST3GAL enzymes has been implicated in several malignancies, driving adhesive and metastatic behavior [6,8,9]. Our previous work further revealed that ST3GAL4 correlates with malignant phenotypes in osteosarcoma [10]. Its knockdown markedly reduces cell proliferation, migration, invasion, and the polarization of M2 tumor-associated macrophages (TAMs) [10]. Moreover, ST3GAL4 facilitates immune evasion in acute myeloid leukemia (AML), and its knockout promotes macrophage-mediated phagocytosis of leukemia cells [11]. Recent evidence further suggests that ST3GAL4 contributes to radiotherapy resistance in triple-negative breast cancer (TNBC) and to targeted-therapy resistance in non-small-cell lung cancer (NSCLC) [12,13]. Together, these studies indicate that ST3GAL4 contributes to cancer progression across multiple tumor types by promoting malignant phenotypes, immune evasion, and therapeutic resistance [10,11,12,13].

Despite these advances, the current understanding of ST3GAL4 remains fragmented, largely limited to single-tumor contexts rather than a unified pan-cancer level. Whether this enzyme exhibits convergent or divergent patterns of genomic alteration, transcriptional activity, protein abundance, or epigenetic regulation across tumor lineages remains unresolved. Furthermore, it is unknown how such molecular heterogeneity relates to patient outcome or to the immunologic composition of the tumor microenvironment at the pan-cancer level. Comprehensive multi-omics analyses integrating genomic, transcriptomic, proteomic, and epigenomic data, together with immune-landscape metrics, offer a path to decipher these broader relationships, yet such approaches have not been systematically applied to ST3GAL4.

Here, we present a pan-cancer analysis of ST3GAL4 across more than thirty tumor types through the integration of datasets from The Cancer Genome Atlas (TCGA), CPTAC, GTEx, and other public resources. We interrogated its genomic, transcriptomic, proteomic, and epigenetic alterations, evaluated prognostic associations, and explored its associations with immune-cell infiltration and glycosylation-associated signaling pathways. Our analyses reveal pervasive but tumor-type-specific dysregulation of ST3GAL4, characterized by distinct molecular signatures and divergent clinical implications. ST3GAL4 expression is further associated with immunosuppressive microenvironmental features, suggesting a mechanistic link between aberrant sialylation and immune escape. Together, these findings establish ST3GAL4 as a cross-tumor regulator at the interface of glycosylation and cancer immunity, providing a conceptual foundation for targeting sialylation pathways in future precision oncology.

## 2. Methods

### 2.1. Pan-Cancer Multi-Omics Expression Analysis and Mutation Profiling

To explore the expression differences in ST3GAL4 between tumor and normal tissues across multiple cancer types, we first performed a differential expression analysis of mRNA levels using the TCGA pan-cancer cohort. The full names and abbreviations of all cancer types are listed in Supplementary Table S1. The TIMER2.0 platform (http://timer.cistrome.org/) (accessed on 13 February 2025) was used for data analysis and visualization [14,15,16]. Under the “Exploration” tab, the “Gene_DE” module was applied by entering the target gene ST3GAL4 to analyze and visualize its mRNA expression levels between tumor and normal tissues. The built-in “edgeR” R package was used to perform statistical differential expression analysis based on raw count data from the TCGA pan-cancer cohort [17]. Cancer types with available matched normal tissues were indicated with a gray background in the output plots.

For cancer types without matched normal tissues in the TCGA pan-cancer cohort (ACC, DLBC, HNSC, LAML, LGG, MESO, OV, SARC, SKCM, TGCT, THYM, UCS, UVM), corresponding normal tissue data from the Genotype-Tissue Expression (GTEx) database were incorporated for differential expression analysis [18]. The normalized TCGA dataset was obtained from the UCSC Xena database (https://xenabrowser.net/) (accessed on 13 February 2025), and batch effects were corrected using the EB++ algorithm before integration with GTEx transcriptomic profiles for subsequent analyses [19].

The GEPIA2 platform (http://gepia2.cancer-pku.cn/) (accessed on 13 February 2025) was used to analyze the mRNA expression levels of ST3GAL4 across different pathological stages [20]. Under the “Expression Analysis” section, the “Stage Plot” module in the “Expression DIY” tab was used by entering the target gene ST3GAL4 and selecting the corresponding cancer types for stage-wise comparison. The platform analyzes transcriptomic data using the transformation log_2_ (TPM + 1).

Proteomic data were obtained from the Clinical Proteomic Tumor Analysis Consortium (CPTAC) and the International Cancer Proteogenome Consortium (ICPC) datasets [21,22,23]. Comparative analyses and visualizations were performed using the University of Alabama at Birmingham Cancer Data Analysis Portal (UALCAN, http://ualcan.path.uab.edu/analysis-prot.html) (accessed on 13 February 2025) [24]. Protein expression data in UALCAN were preprocessed and normalized by the platform: protein expression values downloaded from the CPTAC data portal were log_2_-transformed for each sample, and Z-scores were calculated as standard deviations from the median expression across samples. The expression levels of the ST3GAL4-encoded protein between tumor and normal tissues were analyzed in corresponding cancer types, with all comparisons showing *p* < 0.05.

The cBioPortal database (https://www.cbioportal.org/) (accessed on 13 February 2025) was employed to examine genomic alterations of ST3GAL4 using TCGA Pan-Cancer Atlas datasets [25,26,27]. Mutation and copy number alterations (CANs) data were queried through the “Query by Gene” option, followed by visualization of alteration frequencies across TCGA pan-cancer cohort in the “Cancer Type Summary” module and identification of major mutation sites in the “Mutations module”.

### 2.2. Prognostic Analysis of ST3GAL4 at the Genomic, Transcriptomic, and Epigenetic Levels

At the genomic level, copy number variation (CNV)-based Kaplan–Meier survival analysis was performed using the Tumor Immune Dysfunction and Exclusion (TIDE) platform (http://tide.dfci.harvard.edu/query/) (accessed on 21 February 2025) [28,29]. The original TIDE platform is no longer maintained and has been migrated to the Cancer Immunology Data Engine (CIDE, https://cide.ccr.cancer.gov), which provides updated functionality. Under the “Query Gene” section, the “Copy_Number” function was applied to evaluate the association between CNV status of ST3GAL4 and overall survival (OS). CNV data were standardized and normalized by the TIDE platform: according to previously established criteria [30,31], deep deletions, shallow deletions, diploid status, low-level amplifications, and high-level amplifications were assigned values of −2, −1, 0, +1, and +2, respectively, and subsequently converted to Z-scores for analysis.

At the transcriptomic level, mRNA expression-based survival analysis was conducted using transcriptomic data and corresponding follow-up information from the TCGA pan-cancer cohorts. Patients were divided into high- and low-expression groups according to the median ST3GAL4 mRNA level. Clinical endpoint overall survival (OS) was analyzed with the R package “survival”, using the functions “survfit” for Kaplan–Meier curve generation and “coxph” for hazard ratio estimation across cancer types.

At the epigenetic level, DNA methylation-based survival analysis was also carried out through the TIDE platform under the “Query Gene” section using the “Methylation” function [28,29]. Methylation data were preprocessed and normalized within TIDE: only the probe showing the strongest negative correlation between methylation level and gene expression was retained for each gene represented by multiple probes. The selected probe data were then Z-score-normalized before survival comparison.

### 2.3. Correlation of ST3GAL4 Expression with Genomic Heterogeneity Metrics

Normalized pan-cancer mRNA expression data and corresponding clinical annotations were obtained from the TCGA database through the UCSC Xena platform (https://xenabrowser.net/) (accessed on 21 February 2025). Somatic mutation calls (Level-4 Simple Nucleotide Variation dataset processed by the MuTect2 pipeline) for all TCGA samples were downloaded from the Genomic Data Commons (GDC) portal (https://portal.gdc.cancer.gov/) (accessed on 21 February 2025) [32]. Based on these mutation profiles, tumor mutational burden (TMB) was calculated as the number of somatic mutations per megabase using the “tmb” function of the R package “maftools” [33], and mutant-allele tumor heterogeneity (MATH) scores were derived from the distribution of variant-allele frequencies using the “math.score” function [33].

Microsatellite instability (MSI) status and neoantigen load (NEO) data were integrated from previously published TCGA resources [34,35]: MSI was determined with the MANTIS algorithm [36], and potential neoantigenic peptides were predicted using NetMHCpan v3.0 [37] based on human leukocyte antigen (HLA) genotypes inferred from RNA-seq data using OptiType [38].

Tumor purity, ploidy, homologous recombination deficiency (HRD) scores, and genome-wide loss of heterozygosity (LOH) fractions were retrieved from the TCGA Pan-Cancer Atlas summaries available in UCSC Xena [19]. For each cancer type, the associations between ST3GAL4 expression and the above genomic heterogeneity metrics were evaluated using Spearman’s rank correlation, and two-sided *p* values were adjusted for multiple testing across metrics and cancer types using the Benjamini–Hochberg method. Results with a false discovery rate (FDR) < 0.05 were considered statistically significant.

### 2.4. Correlation of ST3GAL4 Expression with Cancer Stemness

The DNA methylation-based cancer stemness index (DMPsi) was obtained from the TCGA pan-cancer dataset as defined by Malta et al. [39]. Briefly, DMPsi scores were derived using an OCLR (one-class logistic regression) machine-learning model trained on stem cell methylation profiles and applied to TCGA methylation β-value matrices restricted to differentially methylated probes between tumor and normal tissues. Higher DMPsi values indicate a greater degree of tumor dedifferentiation and stem-cell-like features. DMPsi values for each TCGA sample were retrieved from the corresponding pan-cancer resource Sangerbox (http://sangerbox.com/) (accessed on 21 February 2025) [40]. The association between ST3GAL4 expression and DMPsi across cancer types was evaluated using Spearman’s rank correlation, and *p* values were adjusted for multiple testing using the Benjamini–Hochberg method (FDR < 0.05 considered significant).

### 2.5. Association Between ST3GAL4 Promoter Methylation and TIDE-Derived CTL Infiltration Score

The association between ST3GAL4 promoter methylation and cytotoxic T lymphocyte (CTL) infiltration was evaluated using the Tumor Immune Dysfunction and Exclusion (TIDE) platform (http://tide.dfci.harvard.edu/query/) (accessed on 21 February 2025) [28,29], which has since been updated and migrated to the Cancer Immunology Data Engine (CIDE, https://cide.ccr.cancer.gov). Under the “Query Gene” tab, the target gene ST3GAL4 was input, and among the retrieved results, the “Methylation” module was selected for analysis. This module lists promoter methylation correlations of ST3GAL4 across all cancer types in the TCGA pan-cancer cohort. The “CTL Cor” column reports the Spearman correlation coefficients (R values) between ST3GAL4 promoter methylation levels and TIDE-predicted CTL infiltration scores for each cancer type. Clicking on the R values provides scatter plots visualizing these correlations for individual cancers.

### 2.6. Pan-Cancer Correlation Heatmap of ST3GAL4 and m6A Regulator Expression

The correlation between ST3GAL4 expression and RNA modification-related regulator genes was analyzed using the SangerBox platform (http://sangerbox.com/) (accessed on 21 February 2025) [40]. The uniformly processed pan-cancer transcriptomic dataset “TCGA TARGET GTEx” was retrieved from the UCSC Xena database (https://xenabrowser.net/) (accessed on 21 February 2025) [19]. Expression data of ST3GAL4 and 44 RNA modification–related genes, including 10 N1-methyladenosine (m^1^A), 13 5-methylcytosine (m^5^C), and 21 N6-methyladenosine (m^6^A) regulators [41,42,43], were extracted. Samples annotated as “Primary Solid Tumor”, “Primary Tumor”, “Primary Blood Derived Cancer-Bone Marrow”, or “Primary Blood Derived Cancer-Peripheral Blood” were retained, while all normal tissue samples were excluded. Expression values were transformed by log_2_ (x + 0.001) normalization. Pearson correlation coefficients between ST3GAL4 and the RNA modification-related genes were then computed across all retained cancer types, and the correlation matrix was visualized as a heatmap on the SangerBox platform [40].

### 2.7. Identification of Genes Most Correlated with ST3GAL4 Expression at the Transcriptomic Level

To identify genes most positively correlated with ST3GAL4 expression at the transcriptomic level, gene expression data of the TCGA pan-cancer cohort were analyzed. For each cancer type, pairwise correlations between ST3GAL4 mRNA levels and the expression levels of all other genes were calculated based on RNA-seq data using Spearman’s rank correlation. Genes were then ranked according to their correlation coefficients (*R* values), and the top five genes showing the strongest associations with ST3GAL4 expression were selected.

The correlation matrix of ST3GAL4 and the top correlated genes across cancer types was visualized as a heatmap using the TIMER2.0 platform (http://timer.cistrome.org/) (accessed on 21 February 2025) [14,15,16]. In addition, the correlations between ST3GAL4 and each of the top five genes were further validated and visualized as scatter plots using the GEPIA2 platform (http://gepia2.cancer-pku.cn/) (accessed on 21 February 2025) [20].

### 2.8. Protein–Protein Interaction Network and Functional Enrichment Analysis of ST3GAL4-Associated Proteins

The protein–protein interaction (PPI) network of ST3GAL4 was constructed using the STRING database (https://string-db.org/) (accessed on 21 February 2025) [44]. The predicted potential interaction by STRING is based on experimental data, computational prediction, co-expression evidence, and text-mining information from multiple sources [44]. ST3GAL4 was entered as the query gene, and the organism was set to Homo sapiens. Interacting proteins were retrieved with a minimum required interaction score of 0.4 (medium confidence), and the resulting PPI network was visualized directly through the STRING interface.

The interacting proteins identified from the PPI network were further analyzed in the “Analysis” module of STRING to obtain Gene Ontology (GO) enrichment results-including biological process (BP), cellular component (CC), and molecular function (MF) terms-as well as Kyoto Encyclopedia of Genes and Genomes (KEGG) pathway enrichment. In addition, STRING integrates PubMed text-mining information, which was used to summarize the main research areas and biological contexts associated with these ST3GAL4-interacting proteins [44]. The enrichment analyses were performed using the built-in STRING functional enrichment tool, and significance was defined as FDR < 0.05.

### 2.9. Correlation Analysis of ST3GAL4 Expression with Tumor Microenvironment Scores Derived from the ESTIMATE Algorithm

The association between ST3GAL4 expression and tumor microenvironment-related scores was analyzed based on the ESTIMATE algorithm (Estimation of STromal and Immune cells in MAlignant Tumor tissues using Expression data) [45]. The uniformly processed TCGA transcriptomic dataset was downloaded from the UCSC Xena database (https://xenabrowser.net/) (accessed on 21 February 2025) [19]. Expression profiles were preprocessed by log_2_ (x + 0.001) transformation. ImmuneScore, StromalScore, and ESTIMATEScore were computed for each tumor sample using the “ESTIMATE” R package according to the developer’s instructions [45].

Pearson correlation coefficients between ST3GAL4 expression levels and the three ESTIMATE-derived scores were calculated across all cancer types. The pan-cancer correlation results were visualized as bar charts using the SangerBox platform (http://sangerbox.com/) (accessed on 21 February 2025) [40]. For specific cancer types showing the strongest correlations, detailed scatter plots were further generated in SangerBox to illustrate the relationships between ST3GAL4 expression and each of the three scores (ImmuneScore, StromalScore, and ESTIMATEScore) within that specific cancer type [40].

### 2.10. Association of ST3GAL4 Expression with Immunomodulators and Immune/Molecular Subtypes Across Cancers

The associations between ST3GAL4 expression and various immunomodulators were explored using the TISIDB database (http://cis.hku.hk/TISIDB/) (accessed on 21 February 2025) [46]. ST3GAL4 was submitted as the query gene, and within the “Immunomodulator” module, the expression correlations between ST3GAL4 and three classes of immunomodulatory molecules-immunostimulators, immunoinhibitors, and major histocompatibility complex (MHC) molecules-were analyzed across all TCGA cancer types. These immunomodulators were collected and annotated according to published data [47]. The correlation heatmaps displaying ST3GAL4 expression and the three immunomodulator categories across cancers were directly generated on the TISIDB platform.

The “Subtype” module of TISIDB was used to evaluate the distribution differences in ST3GAL4 expression among distinct immune subtypes and molecular subtypes of various cancers in the TCGA cohort. Bar plots illustrating pan-cancer expression differences and violin plots showing expression variation among specific subtypes within selected cancer types were generated by the TISIDB platform [46]. Statistical significance of expression differences among subtypes was assessed using the Kruskal–Wallis test (*p* < 0.05 considered significant).

### 2.11. Association of ST3GAL4 Expression with Immune Checkpoint Genes

Pan-cancer gene expression data were downloaded from the UCSC Xena database (https://xenabrowser.net/) (accessed on 13 February 2026), using the TCGA Pan-Cancer cohort, which had been uniformly processed and normalized. Expression data for ST3GAL4 and 60 immune checkpoint genes (24 inhibitory and 36 stimulatory genes) were extracted [34].

Associations between ST3GAL4 and immune checkpoint genes were analyzed using the SangerBox web platform (http://sangerbox.com/) (accessed on 13 February 2026) based on the TCGA Pan-Cancer RNA-seq dataset [40]. Only tumor samples were included. Expression values were log_2_-transformed within the platform, and Pearson correlation coefficients were computed for each cancer type.

### 2.12. Single-Cell Expression Profiling of ST3GAL4

Single-cell RNA-sequencing (scRNA-seq) data were obtained from the Tumor Immune Single-cell Hub 2 (TISCH2, https://tisch.compbio.cn/home/) (accessed on 13 February 2026), a curated database of tumor microenvironment single-cell transcriptomic datasets [48,49]. The gene ST3GAL4 was queried by selecting the “all cancers” option under the “cancer type” category to retrieve datasets across tumor types. Datasets derived from samples treated with immunotherapy were excluded to minimize therapy-induced transcriptional bias. Among the remaining datasets, representative tumor cohorts with detectable ST3GAL4 expression and available cell-type annotations were included for visualization. ST3GAL4 expression levels across annotated cell populations were extracted and visualized as a heatmap by the TISCH2 platform. Expression values were presented as log (TPM/10 + 1).

### 2.13. Correlation of ST3GAL4 Expression with Immune Cell Infiltration and Immunotherapy Response

The association between ST3GAL4 expression and immune cell infiltration levels across cancers was analyzed using the TIMER2.0 platform (http://timer.cistrome.org/) (accessed on 13 February 2025) [14,15,16]. In the “Immune” module, under the “Gene” tab, ST3GAL4 was submitted as the query gene, and correlations with specific immune cell types were examined using multiple deconvolution algorithms integrated into TIMER2.0. The correlation coefficients were calculated based on TCGA bulk RNA-seq data, and the relationships between ST3GAL4 expression and infiltration levels of selected immune cell subsets were visualized as scatter plots generated by the platform.

The expression distribution of ST3GAL4 between different immunotherapy response groups was further evaluated using the TISMO database (http://tismo.cistrome.org/) (accessed on 21 February 2025) [50]. TISMO integrates in vitro RNA-seq samples from multiple cancer cell lines and in vivo RNA-seq samples from different syngeneic mouse tumor models. The ST3GAL4 expression levels were normalized as log_2_ (TPM + 1) values, and their distributions among different immunotherapy response groups were visualized as bar plots on the TISMO platform [50].

### 2.14. Multiplex Immunohistochemistry Staining and Image Acquisition

Tumor tissues and matched adjacent normal tissues were obtained from Xiangya Hospital of Central South University. Tissue collection and experimental procedures were approved by the Medical Ethics Committee of Xiangya Hospital of Central South University (Approval number: 202303046 and 202401003).

Multiplex immunohistochemistry (mIHC) was performed on formalin-fixed, paraffin-embedded tissue sections from selected tumor types and corresponding adjacent tissues to evaluate ST3GAL4 protein expression and to explore its spatial association with immune cell infiltration, including macrophages and CD4^+^ and CD8^+^ T cells. Tissue sections were dewaxed, rehydrated, and blocked with 5% bovine serum albumin (BSA) to reduce nonspecific binding. The sections were then incubated sequentially with the following primary antibodies: ST3GAL4 (human, 1:150, Proteintech, 13546-1-AP), CD206 (human, 1:400, Cell Signaling Technology, 24595S), CD68 (human, 1:500, Abcam, Cambridge, United Kingdom, ab213363), CD4 (human, 1:200, Cell Signaling Technology, Danvers, MA, USA, 99785T), and CD8 (human, 1:400, Cell Signaling Technology, Danvers, MA, USA, 85336T).

After primary antibody incubation, sections were treated with the corresponding secondary antibodies (RecordBio, Shanghai, China, RC0086Plus-45RM). Nuclear counterstaining was performed using 4, 6-diamidino-2-phenylindole (DAPI), followed by mounting with antifade mounting medium. Multispectral fluorescence images were acquired using an Automatic Multi-label Multispectral Quantitative Pathological Imaging System (VECTRA Polaris, Akoya Biosciences, Marlborough, MA, USA).

The excitation and emission wavelengths for each fluorophore were as follows: DAPI (blue, excitation 330–380 nm, emission 420 nm), CD8/CD206 (cyan, excitation 550 nm, emission 570 nm), CD4/CD68 (red, excitation 590 nm, emission 620 nm), and ST3GAL4 (yellow, excitation 630 nm, emission 690 nm). Image acquisition and visualization of positively stained cells at the single-cell level were performed using Phenochart image analysis software (version 1.0).

A detailed summary table (Supplementary Table S2) listing all analyses, corresponding datasets, platforms, software/tools, and dates of data access is provided in the Supplementary Materials to enhance methodological transparency and reproducibility.

## 3. Results

### 3.1. Pan-Cancer Landscape of ST3GAL4 Expression and Clinical Relevance

**Figure 1:**
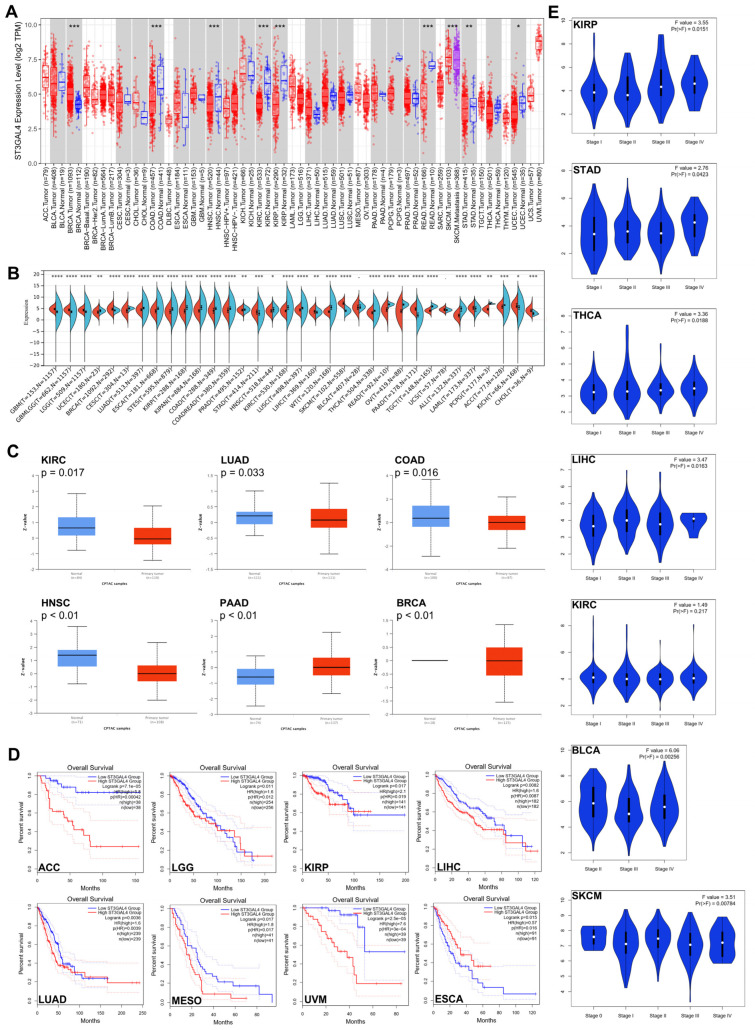
Pan-cancer expression and clinical characteristics of ST3GAL4. (**A**) ST3GAL4 mRNA expression in tumor versus matched normal tissues across TCGA cancer types based on RNA-seq data (FPKM/TPM as provided by TCGA). Statistical significance was evaluated using the Wilcoxon test (* *p* < 0.05, ** *p* < 0.01, *** *p* < 0.001). Red: tumor; Blue: normal tissue; Purple: metastatic tumor. (**B**) ST3GAL4 mRNA expression in TCGA tumor tissues compared with normal tissues, supplemented from the GTEx database (TPM values). Statistical significance was determined using the Wilcoxon test (* *p* < 0.05, ** *p* < 0.01, *** *p* < 0.001, **** *p* < 0.0001). Orange: tumor; Cyan: normal tissue. (**C**) ST3GAL4 protein expression in tumor versus adjacent normal tissues from CPTAC proteomic datasets for KIRC, LUAD, COAD, HNSC, PAAD, and BRCA. Protein abundance represents normalized mass-spectrometry-derived values. (**D**) Overall survival curves for cancer types showing significant differences between high and low ST3GAL4 expression groups in TCGA cohorts. Survival analysis was performed using the Kaplan–Meier method and log-rank test. (**E**) ST3GAL4 mRNA expression across pathological stages in KIRP, STAD, THCA, LIHC, KIRC, BLCA, and SKCM, based on TCGA RNA-seq TPM/FPKM data. Comparisons across stages were performed using the Kruskal–Wallis test.

We first compared ST3GAL4 expression between tumors and matched normal tissues across TCGA cancer types (Figure 1A). ST3GAL4 showed marked dysregulation, being significantly upregulated in BRCA but downregulated in COAD, HNSC, KIRC, KIRP, READ, STAD, and UCEC. As several tumor types lack adequate paired normal tissue, we incorporated normal tissues from GTEx to construct an extended reference cohort (Figure 1B). Using this combined baseline, ST3GAL4 became significantly dysregulated in nearly all cancer types, with some tumors (e.g., GBM, LGG, BRCA) exhibiting prominent upregulation, whereas others (e.g., CESC, LUAD, ESCA) showed clear downregulation. Overall, these analyses indicate that ST3GAL4 is aberrantly expressed across human cancers, with both the direction and magnitude of dysregulation varying by tumor type and reference tissue.

We next assessed whether these transcriptional patterns were reflected at the protein level. Analysis of CPTAC proteomic datasets revealed increased ST3GAL4 protein abundance in PAAD and BRCA, while reduced protein levels were observed in KIRC, LUAD, COAD, and HNSC (Figure 1C), consistent with the RNA-seq findings.

We then evaluated the association between ST3GAL4 expression and overall survival across TCGA cancers (Figure 1D). High ST3GAL4 expression was significantly correlated with poorer survival in ACC, LGG, KIRP, LIHC, LUAD, MESO, and UVM, whereas ESCA displayed an opposite trend. No significant associations were found in the remaining tumor types.

Finally, we examined ST3GAL4 expression across pathological stages for cancers with available staging information (Figure 1E). Several cancers, including KIRP, STAD, THCA, LIHC, BLCA, and SKCM, demonstrated stage-dependent increases in ST3GAL4 expression.

### 3.2. Genomic Alteration Landscape of ST3GAL4 Across Human Cancers

**Figure 2:**
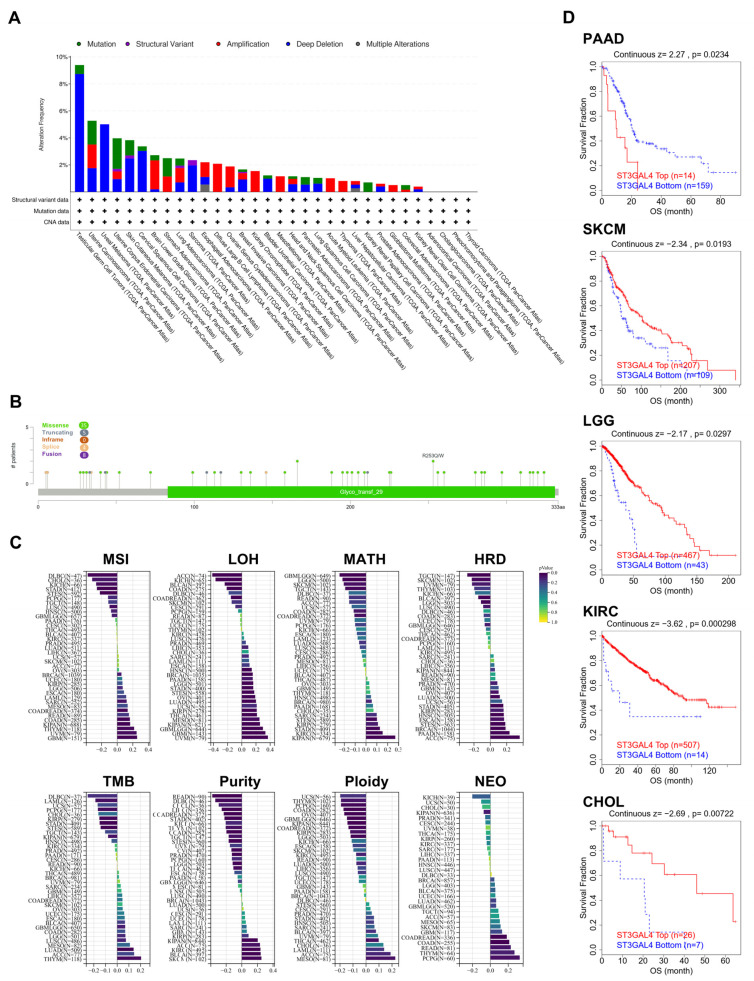
Genomic alterations and genomic instability features associated with ST3GAL4 across cancers. (**A**) Frequency and types of ST3GAL4 genomic alterations across TCGA cancer types, including mutation, structural variant, amplification, deep deletion, and multiple alterations. Data were obtained from cBioPortal (+, positive for the indicated marker). (**B**) Lollipop plot showing the distribution and positions of ST3GAL4 mutations along the coding sequence, annotated by mutation type (missense, truncating, in-frame, splice, fusion). (**C**) Correlations between ST3GAL4 mRNA expression and genomic instability-related features, including microsatellite instability (MSI), loss of heterozygosity (LOH), mutant-allele tumor heterogeneity (MATH), homologous recombination deficiency (HRD), tumor mutation burden (TMB), tumor purity, ploidy, and neoantigen load (NEO), across TCGA cancer types. Correlation coefficients and *p*-values are displayed for each tumor type. (**D**) Overall survival curves for cancer types showing significant differences between ST3GAL4-altered and ST3GAL4-wild-type groups, including PAAD, SKCM, LGG, KIRC, and CHOL. Survival analyses were performed using the Kaplan–Meier method and log-rank test.

We first summarized the genomic alterations of ST3GAL4 across TCGA cancer types (Figure 2A). Overall alteration frequencies were low, generally below 10%, with amplifications being the most frequent event, followed by deep deletions, missense mutations, and structural variants. Tumor types such as TGCT, UCA, UVM, and UCEC displayed relatively higher alteration rates compared with others.

We next mapped mutation sites along the ST3GAL4 coding sequence (Figure 2B). Missense mutations represented the majority of variants, followed by truncating, in-frame, and fusion events. Mutations were broadly distributed, with occasional recurrent sites such as R253Q/W.

We then assessed the relationship between ST3GAL4 expression and genomic instability features across cancers (Figure 2C). ST3GAL4 expression showed tumor-type-dependent correlations with microsatellite instability (MSI), loss of heterozygosity (LOH), mutant allele tumor heterogeneity (MATH), homologous recombination deficiency (HRD), tumor mutation burden (TMB), tumor purity, ploidy, and neoantigen load (NEO), with both the strength and direction of associations varying across cancer types.

We further evaluated the prognostic impact of ST3GAL4 alterations (Figure 2D). In PAAD, ST3GAL4-altered cases exhibited significantly worse overall survival compared with wild-type tumors. In contrast, SKCM, LGG, KIRC, and CHOL showed improved survival in the alteration group. No significant survival differences were observed in other tumor types.

### 3.3. Associations of ST3GAL4 with Cancer Stemness, Immune Cytotoxicity, Methylation, and RNA Modification Regulators

**Figure 3:**
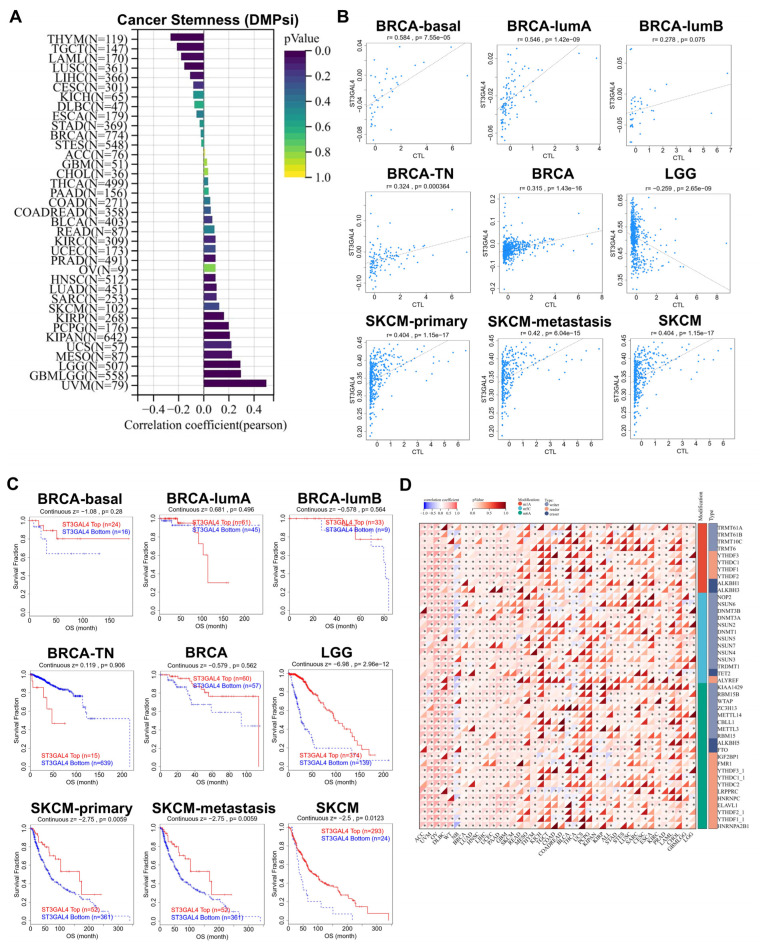
Associations of ST3GAL4 with cancer stemness, cytotoxic T lymphocytes, DNA methylation, and RNA modification regulators. (**A**) Correlation between ST3GAL4 mRNA expression and cancer stemness (DMPsi score) across TCGA cancer types. Pearson correlation coefficients and corresponding *p*-values are shown for each tumor type. (**B**) Scatter plots showing correlations between ST3GAL4 mRNA expression and cytotoxic T lymphocyte (CTL) levels in selected tumor subtypes, including BRCA-basal, BRCA-lumA, BRCA-lumB, BRCA-TN, BRCA overall, LGG, SKCM-primary, SKCM-metastasis, and SKCM. (**C**) Overall survival curves comparing high versus low ST3GAL4 methylation groups in BRCA-basal, BRCA-lumA, BRCA-lumB, BRCA-TN, BRCA overall, LGG, SKCM-primary, SKCM-metastasis, and SKCM. Survival analyses were performed using the Kaplan–Meier method and log-rank test. (**D**) Heatmap of correlation coefficients and *p*-values between ST3GAL4 mRNA expression and regulators of m1A, m5C, and m6A RNA modifications across TCGA cancer types (* *p* < 0.05).

We first evaluated the correlation between ST3GAL4 expression and cancer stemness across TCGA tumor types using the DMPsi score (Figure 3A). ST3GAL4 showed heterogeneous associations with stemness features, with positive correlations observed in tumors such as THYM, TGCT, and LAML, whereas negative correlations were detected in LGG, GBM, and UVM. The direction and magnitude of correlations varied substantially across cancer types.

We next examined the association between ST3GAL4 expression and cytotoxic T lymphocyte (CTL) levels across multiple cancer subtypes (Figure 3B). ST3GAL4 expression showed significant positive correlations with CTL infiltration in BRCA-basal, BRCA-lumA, BRCA-TN, BRCA overall, SKCM-primary, SKCM-metastasis, and SKCM. In contrast, LGG exhibited a significant negative correlation between ST3GAL4 expression and CTL levels. Correlation strengths were generally positive across most tumor subtypes, with LGG representing a notable exception in which ST3GAL4 expression was negatively correlated with CTL levels.

We further examined the prognostic relevance of ST3GAL4 methylation in cancer types showing significant correlations between methylation and gene expression (Figure 3C). In BRCA subtypes, high- and low-methylation groups demonstrated visibly separated survival curves, although the differences did not reach statistical significance. In LGG, higher ST3GAL4 methylation was significantly associated with longer overall survival. Similarly, in SKCM, SKCM-primary, and SKCM-metastasis, the high-methylation group exhibited significantly improved survival compared with the low-methylation group.

Finally, we characterized the relationship between ST3GAL4 expression and RNA modification regulators, including m1A-, m5C-, and m6A-related writers, erasers, and readers (Figure 3D). ST3GAL4 expression showed predominantly positive correlations with these regulators across the majority of cancer types, with many associations reaching statistical significance. Although correlation strengths varied among individual regulators and tumor types, the overall pattern indicated a broadly consistent positive relationship between ST3GAL4 and RNA modification pathways.

### 3.4. Functional Network and Enrichment Analyses of ST3GAL4

**Figure 4:**
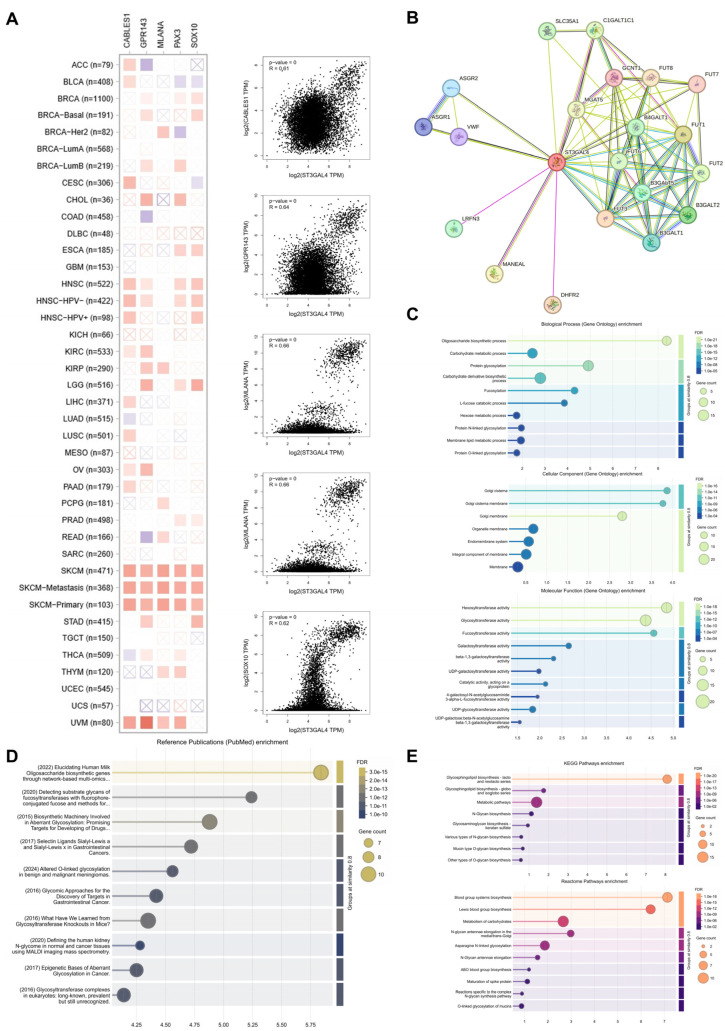
Co-expression profile, protein interaction network, and functional enrichment analyses of ST3GAL4. (**A**) Heatmap showing the top five genes most strongly correlated with ST3GAL4 mRNA expression across TCGA cancer types, accompanied by representative scatter plots illustrating gene-level correlations. Red: positive correlation; light blue: negative correlation; color intensity indicates correlation strength; boxes with “×” denote no statistical significance (*p* > 0.05). (**B**) Protein–protein interaction network for ST3GAL4, constructed using the STRING database, displaying experimentally supported and predicted interacting proteins. (**C**) Gene Ontology (GO) enrichment analysis of ST3GAL4-interacting proteins, including biological process (BP), cellular component (CC), and molecular function (MF) categories. (**D**) Reference publication enrichment (PubMed) of ST3GAL4-associated proteins, showing the top enriched publications based on gene co-occurrence. (**E**) KEGG pathway enrichment and Reactome pathway enrichment analyses of ST3GAL4-interacting proteins.

We first identified the top five genes most strongly correlated with ST3GAL4 across the TCGA pan-cancer cohort: CABLES1, GPR143, MLANA, PAX3, and SOX10 (Figure 4A). These genes showed predominantly positive correlations with ST3GAL4 in most tumor types, with particularly strong associations in melanoma, including both primary and metastatic SKCM. Representative scatter plots illustrate these co-expression patterns.

We next generated a STRING-based protein–protein interaction network (Figure 4B). ST3GAL4 was embedded in a dense interaction module enriched for glycosyltransferases and glycan-processing enzymes, involving multiple members of the FUT, B3GALT, MGAT, and C1GALT1C1 families, along with several additional interacting proteins.

GO enrichment analysis indicated that ST3GAL4-associated proteins were mainly involved in glycan-related biological processes, including oligosaccharide biosynthesis, carbohydrate metabolism, and N-linked and O-linked protein glycosylation (Figure 4C). The enriched cellular components were primarily Golgi-associated membranes, while the dominant molecular functions included a broad spectrum of glycosyltransferase activities, such as hexosyl-, galactosyl-, and fucosyltransferase activity.

Reference publication enrichment further showed that these proteins frequently appeared in studies on aberrant glycosylation, glycosyltransferase biology, selectin ligand pathways, and glycan profiling in normal and cancer tissues, consistent with their roles in glycan biosynthesis (Figure 4D).

Pathway enrichment analyses using KEGG and Reactome demonstrated significant over-representation of pathways related to glycosphingolipid biosynthesis, multiple N-glycan and O-glycan biosynthetic routes, glycosaminoglycan biosynthesis, and blood group antigen biosynthesis, together with additional pathways linked to carbohydrate metabolism and glycan processing (Figure 4E).

### 3.5. Immune Microenvironment Landscape Associated with ST3GAL4 Across Cancers

**Figure 5:**
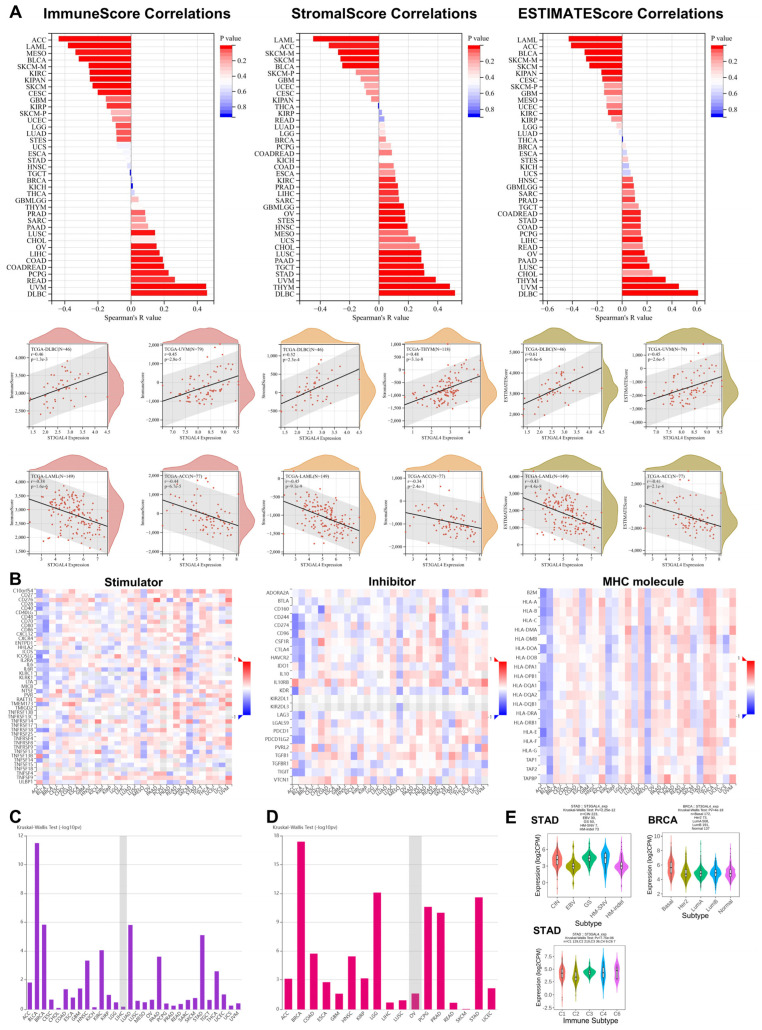
Immune microenvironment features associated with ST3GAL4 expression across cancers. (**A**) Correlations between ST3GAL4 mRNA expression and ESTIMATE-derived immune microenvironment metrics across TCGA cancer types, including ImmuneScore, StromalScore, and ESTIMATEScore. Bar plots show Spearman correlation coefficients for each tumor type. Scatter plots below each bar plot display representative tumor types with the strongest positive and negative correlations for each metric. (**B**) Heatmaps showing correlations between ST3GAL4 mRNA expression and three classes of immunomodulators across TCGA cancers, including immune stimulators, immune inhibitors, and MHC molecules. (**C**) Bar plot summarizing the degree of expression difference in ST3GAL4 across molecular subtypes within each cancer type, quantified using the Kruskal-Walli’s test. (**D**) Bar plot summarizing the degree of expression difference in ST3GAL4 across immune subtypes within each cancer type, quantified using the Kruskal–Wallis test. (**E**) ST3GAL4 mRNA expression in specific cancer subtypes, including molecular and immune subtypes of STAD, and molecular subtypes of BRCA.

We assessed the association between ST3GAL4 expression and multiple immune microenvironment features across TCGA cancers (Figure 5). Correlation analysis using the ESTIMATE algorithm (Figure 5A) showed that ST3GAL4 expression was positively correlated with ImmuneScore, StromalScore, and ESTIMATEScore in several tumor types, with DLBC and UVM exhibiting the strongest positive correlations. In contrast, LAML and ACC showed the most pronounced negative correlations across all three metrics. Representative scatter plots highlight the tumor types with the strongest positive and negative associations.

We next evaluated the relationships between ST3GAL4 expression and three categories of immunomodulators: immune stimulators, immune inhibitors, and MHC molecules (Figure 5B). ST3GAL4 displayed relatively uniform negative correlations with most immunomodulators in ACC and BLCA, whereas ESCA, KIRC, and MESO showed broadly negative correlations between ST3GAL4 expression and nearly all MHC molecules.

We then compared the degree of expression difference in ST3GAL4 across pan-cancer molecular subtypes (Figure 5C) and immune subtypes (Figure 5D). The degree of expression difference in ST3GAL4 varied substantially among molecular subtypes in multiple cancers, reflecting subtype-specific heterogeneity. Similar variation was observed across immune subtypes.

Finally, we examined subtype-specific expression patterns in STAD and BRCA (Figure 5E). In STAD, ST3GAL4 expression differed across both molecular and immune subtypes. Among molecular classifications, HM-SNV displayed the highest expression, whereas EBV showed the lowest levels. Across immune subtypes, ST3GAL4 expression was highest in the C6 subtype and lowest in the C2 subtype. In BRCA, ST3GAL4 expression also differed significantly among molecular subtypes, with the Basal subgroup showing the highest expression, consistent with marked subtype-associated expression gradients.

### 3.6. Immune Infiltration and Immunotherapy Response Associated with ST3GAL4

**Figure 6:**
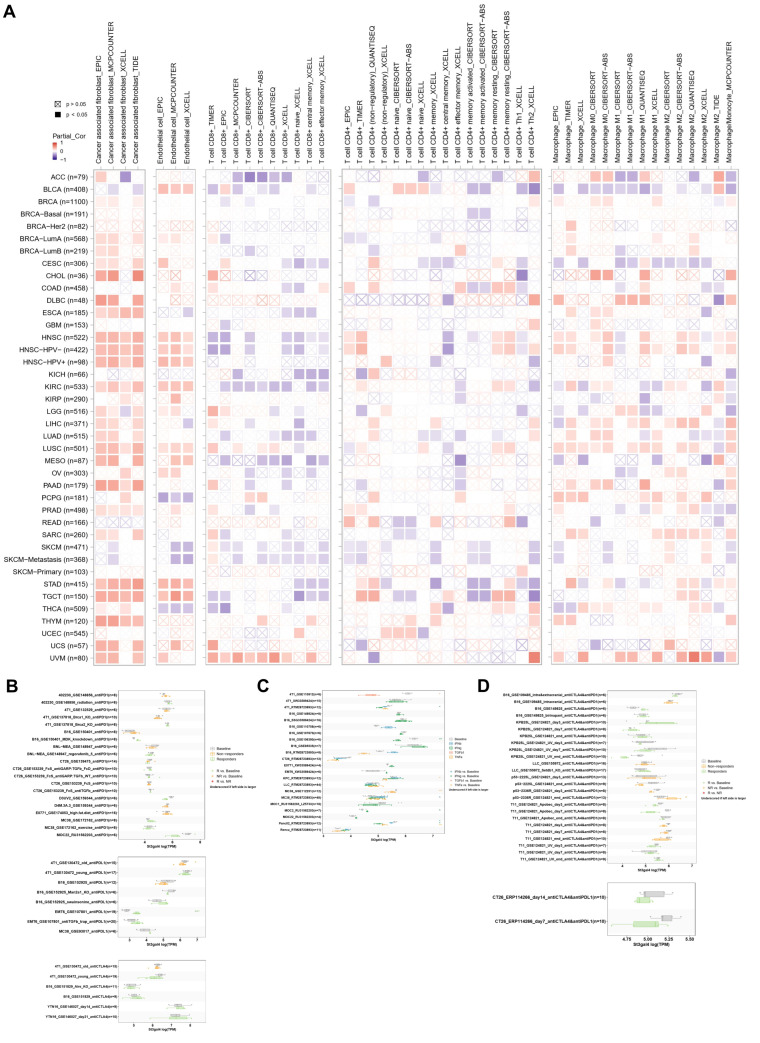
Immune-cell infiltration and cytokine/immunotherapy response associated with ST3GAL4 expression. (**A**) Heatmaps showing correlations between ST3GAL4 mRNA expression and multiple immune cell populations estimated by various deconvolution algorithms across TCGA cancer types, including cancer-associated fibroblasts, endothelial cells, CD8^+^ T cells, CD4^+^ T cells, and multiple macrophage subsets. Correlation coefficients and *p*-values are displayed for each algorithm–tumor-type pair. Filled boxes: *p* < 0.05; boxes with “×”: *p* > 0.05. (**B**) ST3GAL4 expression levels in tumor models treated with anti–PD-1 or anti–PD-L1 monotherapy. Expression differences are shown for baseline, responder, and non-responder groups across multiple independent datasets. (**C**) ST3GAL4 expression following cytokine stimulation across different experimental models. Treatments include IFNβ, IFNγ, TGFβ1, and TNFα. Expression levels after stimulation are compared with baseline controls across datasets derived from breast cancer and melanoma models. (**D**) ST3GAL4 expression levels in mouse tumor models receiving combined immune checkpoint blockade, including anti–CTLA-4 plus anti–PD-1 and anti–CTLA-4 plus anti–PD-L1 therapies. Expression differences are shown for baseline, responder, and non-responder groups.

We analyzed the association between ST3GAL4 expression and immune cell infiltration quantified using multiple deconvolution algorithms across the TCGA pan-cancer cohort (Figure 6A). ST3GAL4 expression showed a broad pattern of positive correlations with tumor-associated fibroblasts (CAFs) across numerous cancer types, with particularly strong associations observed in HNSC and its subtypes, KIRC, STAD, and TGCT. In these same tumors, ST3GAL4 expression was also positively correlated with endothelial cell infiltration, indicating a consistent association with stromal components. In contrast, ST3GAL4 expression displayed widely negative correlations with CD8^+^ T-cell infiltration across most tumor types, with UVM being the primary exception where a positive association was observed. Associations with CD4^+^ T cells and various macrophage subsets (M0/M1/M2) were more heterogeneous, with no unified correlation pattern across deconvolution methods or tumor types.

We next investigated ST3GAL4 expression in preclinical immunotherapy datasets to examine its potential relationship with therapeutic response. In mouse models treated with anti-PD-1 or anti-PD-L1 monotherapy (Figure 6B), ST3GAL4 expression showed model-specific differences: in MOC22 mouse oral squamous cell carcinoma, ST3GAL4 expression was significantly lower in PD-1 responders compared with baseline, whereas in EMT6 mouse mammary carcinoma, ST3GAL4 expression was higher in PD-L1 responders relative to baseline.

Across cytokine-stimulation datasets, ST3GAL4 expression showed a general pattern of decrease following treatment with IFNβ, IFNγ, TGFβ1, or TNFα (Figure 6C). In the GSE110912 breast cancer model, ST3GAL4 expression was significantly lower in the TGFβ1-treated group compared with baseline. In another breast cancer dataset (RTM28723893), ST3GAL4 expression decreased following IFNβ, IFNγ, and TNFα stimulation, with statistical significance observed in the IFNγ-treated group. In the GSE106390 melanoma dataset, IFNγ treatment similarly resulted in significantly reduced ST3GAL4 expression relative to baseline.

We further assessed ST3GAL4 expression under combination checkpoint blockade (Figure 6D). Among multiple treatment contexts, an increase in ST3GAL4 expression was observed only in the non-responder group of the mouse mammary tumor model receiving anti-CTLA-4 plus anti-PD-1 therapy, whereas no consistent response-associated differences were observed in other models or treatment combinations. Additionally, we analyzed the correlation between 60 immune checkpoint genes and ST3GAL4 expression across TCGA cancer types. As shown in Supplementary Figure S1, ST3GAL4 expression exhibited widespread correlations with both inhibitory and stimulatory checkpoint genes in multiple tumor types. Overall, ST3GAL4 expression tended to positively correlate with immune checkpoint genes across cancer types, although heterogeneity was observed. To further delineate the cellular distribution of ST3GAL4 at single-cell resolution, we analyzed representative scRNA-seq datasets from the TISCH database. As shown in Supplementary Figure S2, ST3GAL4 expression was predominantly enriched in malignant cell populations across multiple tumor types. In certain cancers, such as UVM, detectable expression was also observed across several immune subsets, including NK cells, B cells, CD8^+^ T cells, plasma cells, and dendritic cells. However, relative expression levels remained generally higher in malignant compartments compared to most immune populations. In several tumor types, moderate expression was also detected in stromal components such as endothelial cells and fibroblasts.

### 3.7. Multiplex Immunohistochemistry Validation of ST3GAL4 Protein Expression and Immune-Context Features in Human Tumors

**Figure 7:**
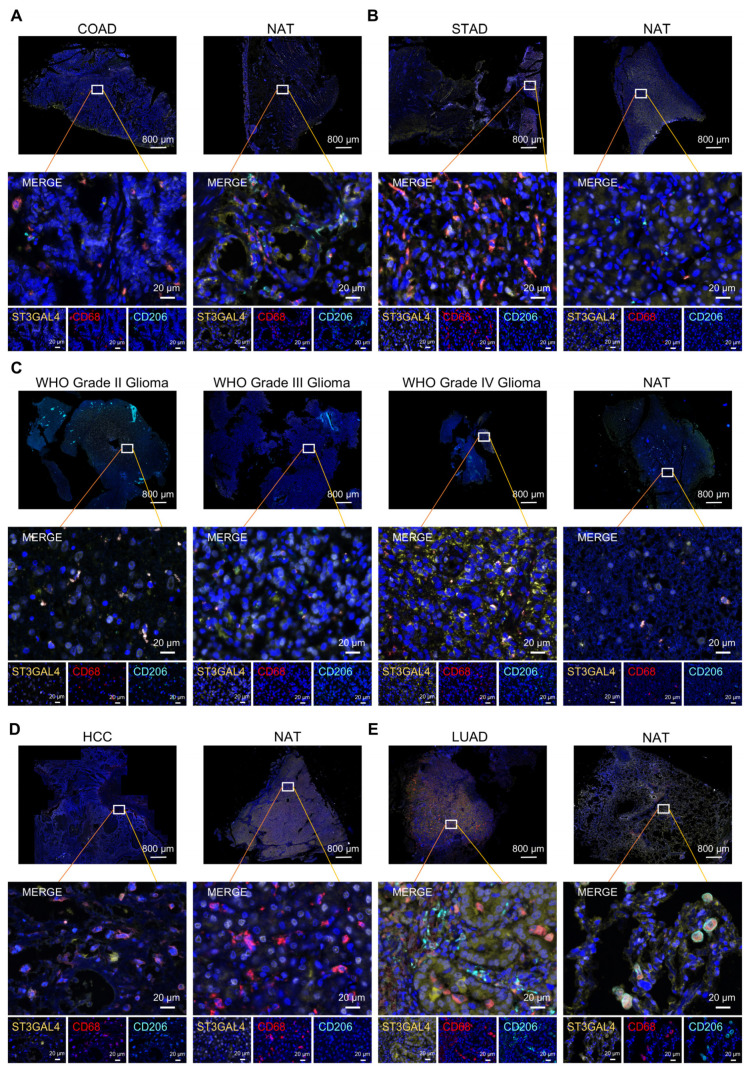
Immunofluorescence staining of ST3GAL4 and macrophage markers in tumor tissues and normal adjacent tissues (NATs). (**A**) Representative multispectral immunofluorescence images of colorectal adenocarcinoma (COAD) and normal adjacent tissues (NATs) stained for ST3GAL4 (yellow), CD68 (red), CD206 (cyan), and nuclei counterstained with DAPI (blue). (**B**) Stomach adenocarcinoma (STAD) and NATs, stained as in (**A**). (**C**) Glioma of WHO grade II-IV and NATs, stained as in (**A**). (**D**) Hepatocellular carcinoma (HCC) and NATs, stained as in (**A**). (**E**) Lung adenocarcinoma (LUAD) and NATs, stained as in (**A**).

**Figure 8:**
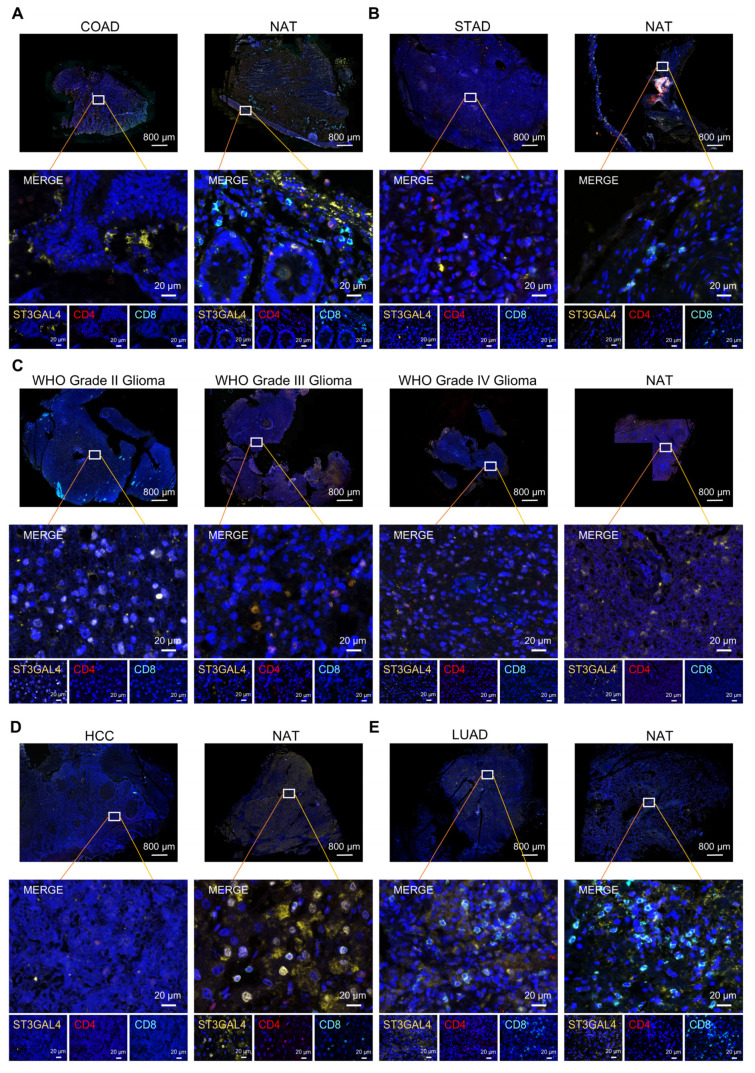
Immunofluorescence staining of ST3GAL4 and T cell markers in tumor tissues and NATs. (**A**) Representative multispectral immunofluorescence images of colorectal adenocarcinoma (COAD) and corresponding NATs stained for ST3GAL4 (yellow), CD4 (red), CD8 (cyan), and nuclei counterstained with DAPI (blue). (**B**) Stomach adenocarcinoma (STAD) and NATs, stained as in (**A**). (**C**) Glioma of WHO grade II-IV and NATs, stained as in (**A**). (**D**) Hepatocellular carcinoma (HCC) and NATs, stained as in (**A**). (**E**) Lung adenocarcinoma (LUAD) and NATs, stained as in (**A**).

Multiplex immunohistochemistry staining was performed to validate ST3GAL4 protein expression in human tumor tissues and corresponding normal tissues adjacent to the tumor (NATs), and to characterize its spatial relationship with macrophage- and T cell-related markers across multiple cancer types (Figure 7, Figure 8 and Figure S3).

In COAD, STAD, glioma (WHO grade II–IV), HCC, and LUAD, tumor sections showed detectable ST3GAL4 signals compared with their corresponding NATs, with ST3GAL4-positive tumor regions exhibiting heterogeneous distribution patterns across cancer types (Figure 7A–E and Figure S3A–E). CD68^+^ macrophages and CD206^+^ M2-like macrophages were consistently detected across multiple tumor types and their matched NATs. However, their spatial distribution, abundance, and infiltration patterns exhibited tumor type-specific characteristics. In COAD, the density of CD68^+^ macrophages was significantly higher in NAT than in tumor tissue, whereas the inverse trend—markedly increased infiltration of CD68^+^ macrophages within tumors—was observed in STAD, HCC, and LUAD. In gliomas, macrophage enrichment was positively correlated with histopathological grade, with lower-grade tumors displaying greater infiltration than their high-grade counterparts (Figure 7 and Figure S3). Notably, across all tumor types except LUAD, elevated ST3GAL4 expression consistently and robustly coincided with increased density of CD206^+^ M2-like macrophages (Supplementary Figure S3), indicating a strong spatial and quantitative association. This consistent co-enrichment supports a potential functional link, wherein ST3GAL4 expression may contribute to the recruitment, retention, or M2-like polarization of tumor-associated macrophages.

To systematically characterize the spatial distribution of T cells within the same tumor type, multiplex immunofluorescence staining for ST3GAL4, CD4, and CD8 was performed on serial tissue sections (Figure 8A–E). Across the tumor types examined, the spatial density of CD4^+^ and CD8^+^ T cells varied between tumor tissues and matched NATs (Figure 8), and notable intratumoral heterogeneity in T cell distribution was also observed. Interestingly, in multiple consecutive tumor sections, regions with high ST3GAL4 expression did not consistently colocalize with areas enriched for CD4^+^ or CD8^+^ T cells, suggesting that ST3GAL4 expression and T cell infiltration may be spatially decoupled at the tissue level (Figure 8 and Figure S3).

Collectively, the immunofluorescence analyses corroborate ST3GAL4 protein detectability in tumor tissues and provide histological context linking ST3GAL4-positive tumor regions with immune-cell–infiltrated microenvironments across COAD, STAD, glioma, HCC, and LUAD.

## 4. Discussion

In this study, we conducted a comprehensive pan-cancer investigation of ST3GAL4, integrating transcriptomic, proteomic, genomic, epigenetic, and immunologic data across multiple tumor lineages. Our findings highlight widespread yet heterogeneous dysregulation of ST3GAL4, together with multi-layered associations implicating this sialyltransferase in tumor progression, stromal remodeling, and immune modulation.

A prominent observation in our analysis is the substantial inter-tumoral variability in ST3GAL4 expression. Certain cancers—including BRCA, GBM, and LGG—exhibited consistent upregulation, whereas others, such as COAD, LUAD, and ESCA, displayed clear downregulation relative to normal tissues. These lineage-specific differences may reflect distinct glycosylation programs or microenvironmental contexts across tumors [4,5]. However, ST3GAL4 represents only one member of the ST3GAL family, and coordinated or compensatory regulation among other isoforms cannot be excluded. A comprehensive analysis of additional ST3GAL isoforms will be necessary to clarify whether the observed effects are specific to ST3GAL4 or reflect broader sialylation network dynamics. Protein-level changes in CPTAC cohorts further aligned with transcriptional alterations, suggesting that ST3GAL4 dysregulation extends beyond mRNA to functional protein abundance.

A growing body of experimental evidence supports a predominantly pro-tumorigenic role for ST3GAL4 across multiple cancer types. ST3GAL4 has been shown to drive immune evasion in acute myeloid leukemia by generating sialylated ligands for the inhibitory receptor Siglec-9, thereby suppressing antitumor immunity [11]. In triple-negative breast cancer, ST3GAL4 upregulation is associated with enhanced α2,3-sialylation, radiotherapy resistance, and inferior survival outcomes [12]. In lung adenocarcinoma, ST3GAL4-mediated sialylation in cancer-associated fibroblasts promotes extracellular matrix secretion and stromal activation, with high infiltration of ST3GAL4-positive CAFs predicting poor overall survival in early-stage patients [51]. Functional studies in pancreatic ductal adenocarcinoma reveal that ST3GAL4 facilitates tumor progression by modulating endoplasmic reticulum stress responses and mitochondrial homeostasis [52]. Similarly, ST3GAL4 has been implicated in glioma development [53] and shown to promote aerobic glycolysis and tumorigenesis in breast cancer [54]. Collectively, these findings support a model in which ST3GAL4 enhances tumor aggressiveness through coordinated regulation of sialylation-dependent signaling, metabolic reprogramming, stromal remodeling, and immune evasion. However, these findings may not uniformly apply across all tumor types, as the biological impact of ST3GAL4 likely depends on tumor lineage, subtype composition, stage distribution, and microenvironmental context.

Importantly, tumor-versus-normal downregulation of ST3GAL4 in bulk datasets does not preclude a context-specific pro-tumor function within subgroups. First, lineage and subtype heterogeneity can generate apparent downregulation at the cohort level while retaining biologically meaningful ST3GAL4-high subsets; this is well recognized in entities with pronounced molecular heterogeneity, such as ESCA and papillary RCC [55,56]. Second, ST3GAL4-mediated hypersialylation may operate in a cell-type–restricted manner and thereby influence progression despite modest or reduced tumor-cell bulk expression. Consistent with this, recent lung cancer studies link ST3GAL4 to aggressive phenotypes and treatment resistance in specific models [13,51]. More broadly, aberrant sialylation is known to modulate adhesion, signaling and immune recognition, and its functional output can vary with the tumor microenvironment and the available Siglec axes [57]. These considerations suggest that the clinical association of ST3GAL4 may depend on subtype composition, microenvironmental context, and compartment-specific expression rather than simply on the direction of tumor-versus-normal differential expression.

Our immune-related analyses revealed several notable associations. ST3GAL4 expression showed strong positive correlations with CAF and endothelial infiltration across multiple tumor types, suggesting a potential role in shaping stromal-enriched microenvironments. This is consistent with the known involvement of aberrant sialylation in extracellular matrix remodeling and stromal activation [51]. Conversely, we observed broadly negative associations between ST3GAL4 and CD8^+^ T-cell infiltration, except in UVM. Given that sialylated glycans can bind inhibitory Siglec receptors on T cells and NK cells, dampening cytotoxic activity [11], these findings raise the possibility that elevated ST3GAL4 contributes to immune suppression in selected tumors. Nonetheless, causal relationships remain to be experimentally validated.

We also observed robust correlations between ST3GAL4 and multiple RNA modification regulators, including m1A-, m5C-, and m6A-related genes. Cross-talk between glycosylation and epitranscriptomic regulation has been increasingly recognized [58], and our results suggest that ST3GAL4 may participate in broader transcriptional or post-transcriptional regulatory programs that contribute to tumor plasticity. Similarly, the context-dependent correlations between ST3GAL4 and cancer stemness scores support a potential link between sialylation pathways and tumor dedifferentiation, though the underlying mechanisms remain unclear.

Clinically, ST3GAL4 expression demonstrated strong prognostic relevance in several cancers, most notably ACC, LGG, KIRP, LIHC, LUAD, MESO, and UVM. The opposite trend observed in ESCA highlights the lineage specificity of ST3GAL4’s functional roles. Alteration-based survival analyses further revealed divergent prognostic implications, with ST3GAL4 alterations portending worse outcomes in PAAD but improved outcomes in SKCM, LGG, KIRC, and CHOL. These observations emphasize the complexity of ST3GAL4 biology and suggest that its prognostic impact may depend on distinct selective pressures or microenvironmental conditions across tumor types.

Specifically, such context dependency may partly arise from tumor subtype heterogeneity: for instance, TCGA has demonstrated that ESCA comprises biologically distinct entities (ESCC vs. EAC) with divergent molecular programs and clinical behaviors, which can confound bulk-level expression patterns and prognosis when subtypes are aggregated [56]. Likewise, KIRP is heterogeneous with different stage distributions and outcomes, indicating that subtype and stage composition can influence survival associations of individual genes [55]. In addition, stage-specific remodeling of glycosylation programs is plausible because glycans/glycosylation participate in multiple steps of tumor progression and metastasis-related phenotypes [59,60]. Finally, immune microenvironment differences and glycosylation-dependent functional switching may contribute, as altered tumor sialylation can modulate cell–cell interactions and immune recognition, and the sialic acid–Siglec axis is increasingly recognized as an immunoregulatory pathway in cancer [61,62].

Our STRING network linked ST3GAL4 with multiple glycosyltransferases (e.g., FUT, B3GALT, MGAT) involved in the biosynthesis of sialylated Lewis-type epitopes. Notably, sialyl Lewis A (sLeA), also known as CA19-9, has been extensively reported as a clinically used glycan marker in gastrointestinal malignancies, particularly pancreatic cancer, and is also described in other GI tumor contexts (e.g., gastric/colorectal/biliary) [63,64]. Beyond its value as a biomarker, tumor-associated sLeA expression has been associated with metastatic behavior and adverse clinical outcomes in patient-derived analyses, supporting its functional relevance in cancer progression [65]. In esophageal squamous cell carcinoma, sLeA expression has also been reported to correlate with hematogenous recurrence after curative resection [66]. Hence, these prior observations provide clinical context suggesting that the ST3GAL4-centered glycosylation network identified here may be mechanistically linked to sLeA-related tumor phenotypes in specific cancer types, warranting future glycomic and functional validation.

Our pathway enrichment analyses provide additional insight into the functional landscape associated with ST3GAL4. The enrichment of glycosphingolipid and glycan biosynthetic pathways—including N-glycan, O-glycan, and glycosaminoglycan routes—is consistent with the biochemical activities of ST3GAL4 and its interacting partners. These pathways profoundly influence receptor stability, ligand binding, and immune recognition [67], offering plausible biological explanations for the multi-dimensional associations observed. Specifically, one potential hypothesis is that increased α2,3-sialylation enhances sialoglycan ligands that engage inhibitory Siglec receptors, thereby promoting immune suppression in the tumor microenvironment [68,69]. Notably, ST3GAL4 was reported to drive immune evasion in AML by increasing inhibitory Siglec-9 ligands, providing proof-of-principle support for an ST3GAL4–Siglec axis [11]. In addition, tumor-associated hypersialylation has been linked to T-cell dysfunction, exhaustion and immunosuppression via Siglecsialoglycan interactions in the microenvironment [57,70]. Beyond the Siglec axis, cytokine-driven immune programs may intersect with glycosylation remodeling: IFN-γ can induce PD-L1 via JAK/STAT–IRF1 signaling, and PD-L1 stability is regulated by N-glycosylation, suggesting a potential interface between ST3GAL4-related glycosylation and checkpoint regulation [71,72]. Moreover, TGF-β stimulation has been shown to reshape cellular glycomes with increased sialylation and altered sialyltransferase expression, supporting a possible link between ST3GAL4 activity and immunosuppressive cytokine contexts [73,74]. Collectively, these observations suggest that ST3GAL4 may contribute to immune evasion through the sialic acid–Siglec and cytokine/checkpoint-associated pathways, which require further functional validation.

We also explored immunotherapy-related datasets to examine whether ST3GAL4 expression may shift in response to immune activation. ST3GAL4 downregulation following cytokine stimulation—particularly IFNγ—is notable, as IFNγ is a key effector of antitumor immunity. Similarly, ST3GAL4 expression differed between responders and non-responders in select checkpoint blockade models, although findings were not consistent across datasets. These preliminary results suggest that ST3GAL4 expression may be sensitive to immune signaling states, but further validation in clinical cohorts will be required.

From a translational perspective, the immunomodulatory implications of ST3GAL4-mediated sialylation raise the possibility that ST3GAL4-high tumors may exhibit a sialoglycan-enriched, immune-suppressive phenotype potentially relevant to immunotherapy response. While direct clinical evidence linking ST3GAL4 to immune checkpoint blockade (ICB) response remains limited, multiple studies support a rationale that elevated tumor sialylation is associated with immunosuppressive states and can constrain ICB efficacy, and that targeting tumor sialylation may repolarize tumor-associated macrophages and enable more effective checkpoint therapy [70,75]. Mechanistically, ST3GAL4 has been implicated in generating inhibitory Siglec ligands and in modulating PD-L1 α2,3-sialylation, suggesting that ST3GAL4-high tumors could plausibly exhibit a sialoglycan-enriched, immunosuppressive microenvironment that contributes to therapeutic resistance [11,76]. Importantly, glyco-immune checkpoint strategies are now entering clinical evaluation; for example, a first-in-class sialidase-based agent (E-602) has been tested in combination with PD-1 blockade in PD-(L)1–resistant solid tumors, and patient stratification by tumor sialoglycan levels has been incorporated, supporting the feasibility of glycosylation-targeted combination therapy paradigms [77]. These observations support evaluating ST3GAL4 not only as a prognostic marker but also as a candidate biomarker to prioritize patients for glycosylation/Siglec-axis–targeted strategies combined with ICB, pending prospective clinical validation.

Multiplex immunofluorescence analyses provided tissue-level validation of ST3GAL4 protein expression across multiple tumor types and their corresponding NATs. ST3GAL4 signals were detectable within tumor regions and were frequently observed in macrophage-enriched areas marked by CD68 and CD206, supporting an association between ST3GAL4 expression and macrophage-infiltrated tumor microenvironments. In contrast, staining for CD4 and CD8 highlighted substantial heterogeneity in T-cell distribution across tumor types and within individual tumor sections, with ST3GAL4-positive regions not uniformly overlapping with T-cell-dense areas. These histological observations are consistent with the pan-cancer computational analyses, suggesting that ST3GAL4 expression is linked to immune-context features rather than reflecting a single immune cell population. Together, the immunofluorescence results serve as an orthogonal validation of the bioinformatic findings and support the relevance of ST3GAL4 as a tumor-associated molecule embedded within immune-modulated tumor ecosystems. The multiplex immunohistochemistry analysis was performed in only a representative set of tumor types (COAD, STAD, glioma, HCC, and LUAD) to provide exploratory validation across distinct tissue lineages and molecular contexts. These tumor types were selected to encompass gastrointestinal, hepatic, pulmonary, and central nervous system malignancies, thereby reflecting diverse glycosylation and immune microenvironmental backgrounds. This panel does not comprehensively cover all cancer types analyzed in the pan-cancer framework. Rather, it serves as a proof-of-concept validation. Future studies with expanded cohorts will be required to systematically evaluate ST3GAL4-associated spatial immune patterns across additional tumor entities.

The spatial immunohistochemical patterns were not entirely concordant with the immune associations inferred from bulk transcriptomic analyses. Such differences may partly arise from the restricted set of tumor types examined, variability in tissue section selection, and the inherently localized nature of histological sampling compared with cohort-level transcriptomic profiling. The incorporation of single-cell transcriptomic profiling helps clarify the predominant cellular source of ST3GAL4 expression and provides a conceptual link between transcriptomic correlations and tissue-level observations (Supplementary Figure S2). Nevertheless, discrepancies across analytical levels may arise from tumor heterogeneity, spatial compartmentalization, and context-dependent glycosylation programs that are not fully captured by bulk deconvolution approaches. Further mechanistic and spatially resolved investigations will be necessary to define the precise relationship between ST3GAL4 expression and immune microenvironment organization.

Although this study provides a multi-omic, macro-scale view of ST3GAL4 dysregulation across human cancers, its intent was exploratory in nature. Motivated by our team’s prior observations implicating sialyltransferase activity in osteosarcoma [10], we sought to determine whether ST3GAL4 exhibited broader cross-cancer patterns and whether these patterns aligned with known glycosylation-associated tumor behaviors. Accordingly, our analyses emphasize systematic landscape-level characteristics rather than tumor-specific mechanistic dissection. Because the datasets used here are predominantly bulk-derived, the cellular sources and functional consequences of ST3GAL4 expression cannot yet be resolved, and the immunologic and stromal associations identified remain correlative. While our immunofluorescence validation confirms ST3GAL4 protein expression in selected tumor tissues, clarifying how ST3GAL4-mediated sialylation shapes stromal architecture, antigen-processing pathways, or the functional state of cytotoxic lymphocytes will require deeper mechanistic studies in defined tumor contexts. Future work should also address whether ST3GAL4 integrates metabolic, glycan-remodeling, and immune-regulatory programs into a unified axis supporting tumor progression or immune evasion, and whether its expression or glycosylation patterns hold predictive value in clinically annotated immunotherapy cohorts.

In summary, our multi-omics pan-cancer analysis reveals that ST3GAL4 is a biologically and clinically relevant molecule with diverse context-dependent roles across human cancers. These findings provide a foundation for future mechanistic and translational studies aimed at elucidating the functional significance of ST3GAL4 in tumor progression and immune regulation.

## Data Availability

The original contributions presented in this study are included in the Methods section. Further inquiries can be directed to the corresponding author.

## Supplementary Materials

The following supporting information can be downloaded at: https://www.mdpi.com/article/10.3390/biomedicines14040766/s1, Figure S1: Correlation between ST3GAL4 Expression and Immune Check-point Genes Across TCGA Cancer Types. Heatmap showing the correlation between ST3GAL4 expression and immune checkpoint genes across TCGA cancer types. Columns represent tumor types and rows represent checkpoint genes. Correlation coefficients were calculated using Pearson analysis. Red indicates positive correlation and blue indicates negative correlation. *p* < 0.05 was considered statistically significant; Figure S2: Single-cell expression landscape of ST3GAL4 across representative tumor types. Heatmap showing ST3GAL4 expression across major cell populations in representative tumor types using publicly available single-cell RNA sequencing datasets from the TISCH database. Rows represent individual tumor datasets and columns denote annotated cell types. Expression values are presented as log (TPM/10 + 1; Figure S3: Statistical analysis of immunofluorescence staining of tumor tissues and NATs. Quantitative metrics included the relative mean fluorescence intensity (MFI) of ST3GAL4, the percentage of CD68^+^ cells among total cells, the percentage of CD206^+^ cells within the CD68^+^ population, and the percentages of CD4^+^ and CD8^+^ T lymphocytes among total cells. The percentage of CD68^+^ cells was calculated by dividing the number of CD68^+^ cells by the total number of DAPI+ nuclei in the same field. (A) COAD. (B) STAD. (C) Glioma. (D) HCC. (E) LUAD; Table S1: Abbreviations of cancer types; Table S2: Summary of datasets and analytical parameters.
