# Supplementary material for: Systematic Pan-Cancer Characterization of ST3GAL4 Reveals Its Prognostic and Immunologic Associations
Source: Biomedicines. 2026 Mar 27;14(4):766. doi: 10.3390/biomedicines14040766 (PMC13113861; doi:10.3390/biomedicines14040766)
Supplement: Supplementary file 1 [file biomedicines-14-00766-s001.zip › biomedicines-4136023-supplementary/Supplementary Files/Supplementary Table S1.pdf]

Supplementary Table S1. Abbreviations of cancer types.

Cancer type	Definition
AML	Acute myeloid leukemia
ACC	Adrenocortical carcinoma
BLCA	Bladder urothelial carcinoma
LGG	Brain lower-grade glioma
BRCA	Breast invasive carcinoma
CESC	Cervical squamous cell carcinoma and endocervical adenocarcinoma
CHOL	Cholangiocarcinoma
COAD	Colon adenocarcinoma
ESCA	Esophageal carcinoma
GBM	Glioblastoma multiforme
HNSC	Head and neck squamous cell carcinoma
KICH	Kidney chromophobe
KIRC	Kidney renal clear cell carcinoma
KIRP	Kidney renal papillary cell carcinoma
LIHC	Liver hepatocellular carcinoma
LUAD	Lung adenocarcinoma
LUSC	Lung squamous cell carcinoma
DLBC	Lymphoid neoplasm diffuse large B-cell lymphoma
MESO	Mesothelioma
OV	Ovarian serous cystadenocarcinoma
PAAD	Pancreatic adenocarcinoma
PCPG	Pheochromocytoma and paraganglioma
PRCA	Prostate carcinoma
PRAD	Prostate adenocarcinoma
READ	Rectum adenocarcinoma
SARC	Sarcoma
SKCM	Skin cutaneous melanoma
STAD	Stomach adenocarcinoma
TGCT	Testicular germ cell tumors
THYM	Thymoma
THCA	Thyroid carcinoma
UCA	Uterine carcinosarcoma
UCEC	Uterine corpus endometrial carcinoma
UVM	Uveal melanoma
