# Supplementary material for: Systematic Pan-Cancer Characterization of ST3GAL4 Reveals Its Prognostic and Immunologic Associations
Source: Biomedicines. 2026 Mar 27;14(4):766. doi: 10.3390/biomedicines14040766 (PMC13113861; doi:10.3390/biomedicines14040766)
Supplement: Supplementary file 1 [file biomedicines-14-00766-s001.zip › biomedicines-4136023-supplementary/Supplementary Files/Supplementary Table S2.pdf]

Supplementary Table S2. Summary of datasets and analytical parameters

Analysis	Dataset	Sample size	Platform	Software	Version	Date
Differential expression analysis of mRNA	TCGA Pan-Cancer cohort	As implemented in TIMER2.0	RNA-seq	TIMER2.0	Online release	Feb 2025
Differential expression analysis of mRNA with normal tissue	TCGA Pan-Cancer & GTEx	As provided by UCSC Xena	RNA-seq	UCSC Xena	Online release	Feb 2025
Expression of mRNA across pathological stages	TCGA tumor samples	As implemented in GEPIA2	RNA-seq	GEPIA2	Online release	Feb 2025
Differential protein expression analysis	CPTAC & ICPC datasets	As implemented in UALCAN	LC–MS/MS	UALCAN	Online release	Feb 2025
Genomic alteration analysis (mutation & CNA)	TCGA Pan-Cancer Atlas	As implemented in cBioPortal	WES	cBioPortal	Online release	Feb 2025
Survival analysis based on CNV data	As implemented in TIDE	See corresponding figures	CNV	TIDE	Online release	Feb 2025
Survival analysis based on mRNA data	TCGA Pan-Cancer cohort	See corresponding figures	RNA-seq	R software	R v 4.1.2	Feb 2025
Survival analysis based on methylation data	As implemented in TIDE	See corresponding figures	Methylation array	TIDE	Online release	Feb 2025
Pan-cancer mRNA expression data acquisition	TCGA Pan-Cancer cohort	As provided by UCSC Xena	RNA-seq	UCSC Xena	Online release	Feb 2025
TMB and MATH score calculation	TCGA (Level-4 SNV, MuTect2)	As provided by GDC portal	WES	R software	R v 4.1.2	Feb 2025
MSI and NEO analysis	Published TCGA-based resources	As provided by the publications	WES and RNA-seq	MANTIS; NetMHCpan	Online release	Feb 2025
Genomic heterogeneity correlation analysis	TCGA Pan-Cancer Atlas	As provided by UCSC Xena	WES and RNA-seq	R software	R v 4.1.2	Feb 2025
DMPsi correlation analysis	TCGA pan-cancer DMPsi	As provided by the publication	Methylation array	Sangerbox	Online release	Feb 2025
Promoter methylation–CTL infiltration correlation	As implemented in TIDE	As implemented in TIDE	Methylation &RNA-seq	TIDE	Online release	Feb 2025
RNA modification regulator correlation analysis	TCGA TARGET GTEx	As implemented in UCSC Xena	RNA-seq	Sangerbox	Online release	Feb 2025
Transcriptomic correlation analysis (top correlated genes)	TCGA Pan-Cancer cohort	Cancer-type specific	RNA-seq	R; TIMER2.0; GEPIA2	R v 4.1.2	Feb 2025
PPI network and functional enrichment analysis	STRING database (Homo sapiens)	Not applicable	Not applicable	STRING	Online release	Feb 2025
Tumor microenvironment score correlation	TCGA Pan-Cancer cohort	Cancer-type specific	RNA-seq	SangerBox	Online release	Feb 2025
Immunomodulator association analysis	As implemented in TISIDB	Cancer-type specific	RNA-seq	TISIDB	Online release	Feb 2025
Immune checkpoint gene correlation analysis	TCGA Pan-Cancer cohort	Cancer-type specific	RNA-seq	SangerBox	Online release	Feb 2026
Single-cell expression profiling	As implemented in TISCH2	Dataset-specific	scRNA-seq	TISCH2	Online release	Feb 2026
Immune cell infiltration correlation analysis	TCGA Pan-Cancer cohort	As implemented in TIMER2.0	RNA-seq	TIMER2.0	Online release	Feb 2025
Immunotherapy response–associated expression analysis	As implemented in TISMO	As implemented in TISMO	RNA-seq	TISMO	Online release	Feb 2025
