# Supplementary figures and images for: Systematic Pan-Cancer Characterization of ST3GAL4 Reveals Its Prognostic and Immunologic Associations
Source: Biomedicines. 2026 Mar 27;14(4):766. doi: 10.3390/biomedicines14040766 (PMC13113861; doi:10.3390/biomedicines14040766)

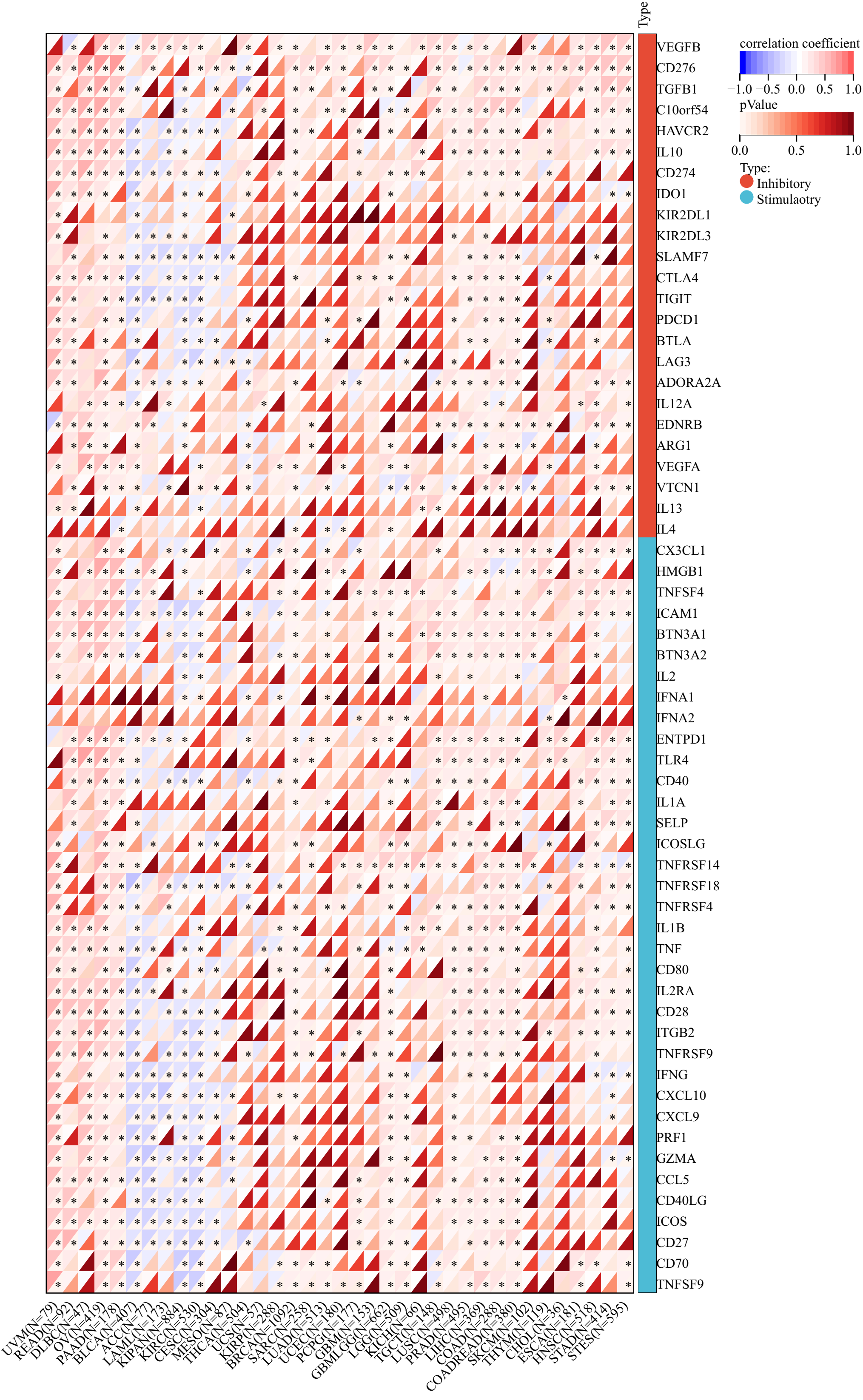

Supplement: Supplementary file 1 [file biomedicines-14-00766-s001.zip › biomedicines-4136023-supplementary/Supplementary Files/Supplementary figure S1.pdf]

ST3GAL4

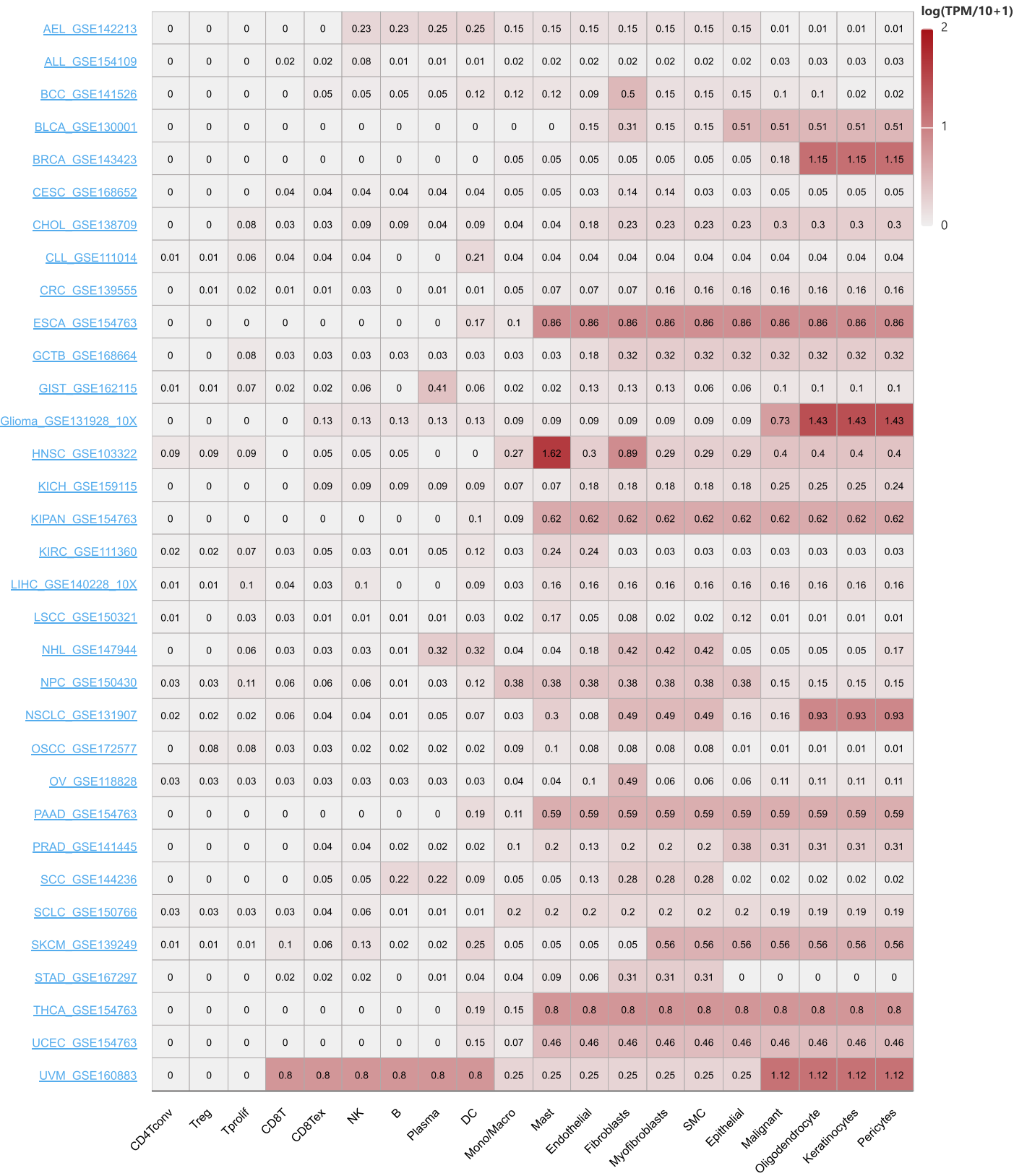

Supplement: Supplementary file 1 [file biomedicines-14-00766-s001.zip › biomedicines-4136023-supplementary/Supplementary Files/Supplementary figure S2.pdf]

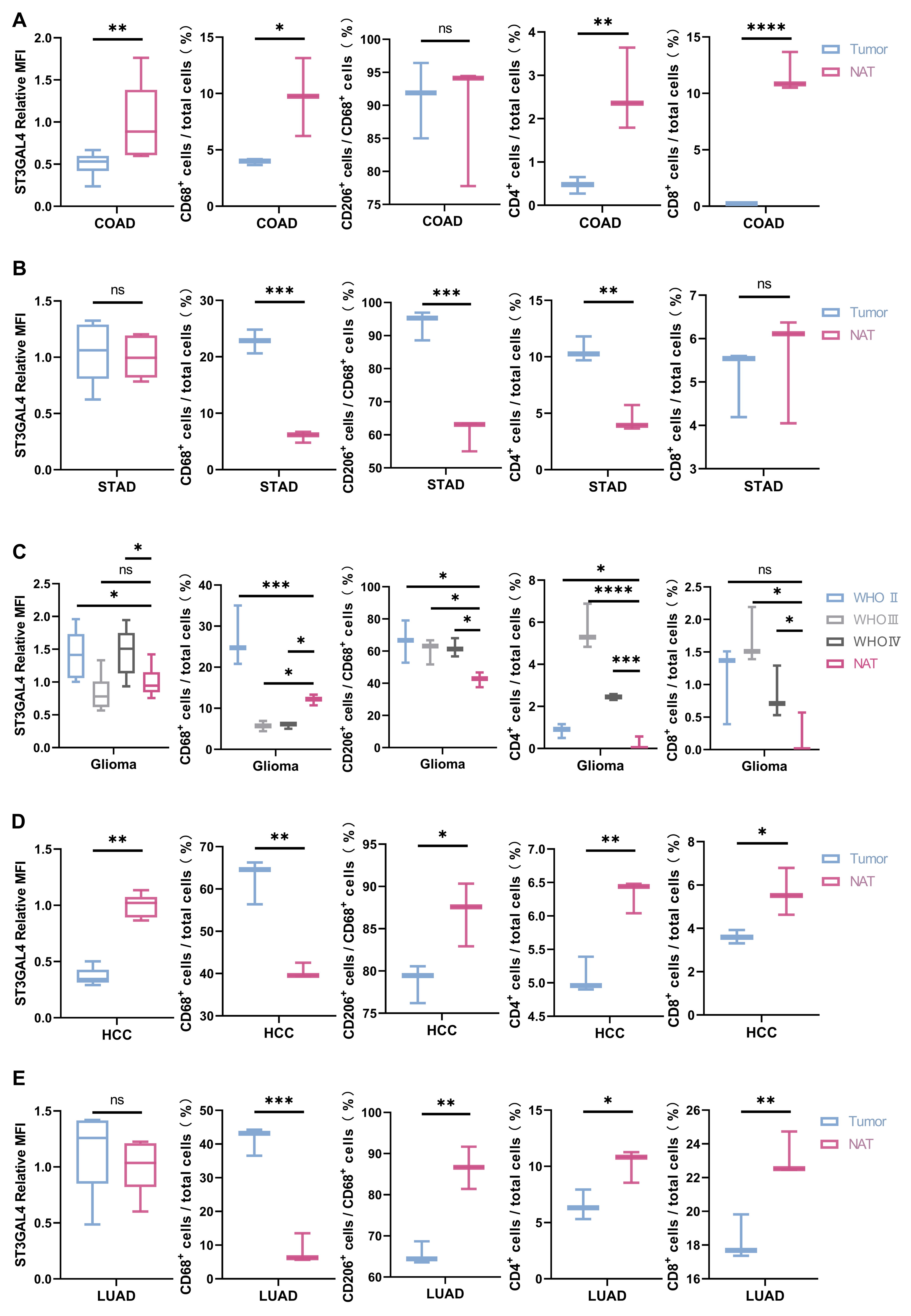

Supplement: Supplementary file 1 [file biomedicines-14-00766-s001.zip › biomedicines-4136023-supplementary/Supplementary Files/Supplementary figure S3.tif]
